# A Rapid Growth-Independent Antibiotic Resistance Detection Test by SYBR Green/Propidium Iodide Viability Assay

**DOI:** 10.3389/fmed.2018.00127

**Published:** 2018-05-03

**Authors:** Jie Feng, Rebecca Yee, Shuo Zhang, Lili Tian, Wanliang Shi, Wen-Hong Zhang, Ying Zhang

**Affiliations:** ^1^Department of Molecular Microbiology and Immunology, Bloomberg School of Public Health, Johns Hopkins University, Baltimore, MD, United States; ^2^Beijing Research Institute for Tuberculosis Control, Beijing, China; ^3^Key Laboratory of Medical Molecular Virology, Department of Infectious Diseases, Huashan Hospital, Shanghai Medical College, Fudan University, Shanghai, China

**Keywords:** antibiotic resistance, antimicrobial susceptibility testing, SYBR Green/PI availability assay, *Mycobacterium tuberculosis*, *Staphylococcus aureus*, CRE bacteria

## Abstract

Antibiotic-resistant bacteria have caused huge concerns and demand innovative approaches for their prompt detection. Current antimicrobial susceptibility tests (AST) rely on the growth of the organisms which takes 1–2 days for fast-growing organisms and several weeks for slow growing organisms. Here, we show for the first time the utility of the SYBR Green I/propidium iodide (PI) viability assay for rapidly identifying antibiotic resistance in less than 30 min for major, antibiotic-resistant, fast-growing bacteria, such as *Staphylococcus aureus, Escherichia coli, Klebsiella pneumoniae*, and *Acinetobacter baumannii* for bactericidal and bacteriostatic agents and in 16 h for extremely rapid detection of drug resistance for isoniazid and pyrazinamide in slow-growing *Mycobacterium tuberculosis*. The SYBR Green I/PI assay generated rapid and robust results in concordance with traditional AST methods. This novel growth-independent methodology changes the concept of the current growth-based AST and may revolutionize current drug susceptibility testing for all cells of prokaryotic and eukaryotic origin and, subject to further clinical validation, may play a major role in saving lives and improving patient outcomes.

## Introduction

A rising public health concern is the increasing emergence and spread of antibiotic-resistant bacteria (1–5). To combat antibiotic resistance, health-care providers are encouraged to minimize the unnecessary usages of antibiotics (6, 7). Hence, laboratory antibiotic or drug susceptibility testing results are imperative in providing health-care providers the knowledge to tailor the appropriate treatment to each individual (8, 9). However, currently, the common drug susceptibility tests (DST) all rely on growth of the organisms and take at least 18–24 h for non-fastidious organisms (10) and can be up to 1 month for fastidious slow growing organisms like *Mycobacterium tuberculosis* (11) before any information can be obtained about the pathogen’s susceptibility or resistance to a particular drug, putting the patient’s life at risk with potential worsened disease outcome and increased spread of antibiotic-resistant organisms. Here, we developed a novel, growth-independent antibiotic susceptibility testing methodology that can produce rapid and robust results in less than 24 h for both slow and fast-growing organisms.

The current antimicrobial susceptibility tests (AST) such as the Kirby–Bauer disk diffusion test or broth dilution method to determine a drug’s minimum inhibitory concentration (MIC) provide accurate results, but the major drawback of these tests is the lengthy time (at least 16 h) required until results are produced even for non-fastidious organisms (10). Recently, a MALDI Biotyper antibiotic susceptibility test rapid assay (MBT-ASTRA) was developed and showed high sensitivity against *M. tuberculosis* but the turnaround time for drug susceptibility results still takes at least 6 days after the cultures flag positive (12). The development of the automated VITEK^®^2 and microdroplet growth assay using MALDI-TOF produced high sensitivity for AST, but these tests are still growth dependent and take at least 12 and 4 h, respectively (13, 14). In the case of multidrug-resistant tuberculosis and extensively drug-resistant TB (XDR-TB), a period of 2–6 weeks is required to obtain drug susceptibility testing results, which increases the risk of the development and spread of drug-resistant TB (15). The current DST for pyrazinamide (PZA), a unique sterilizing persister drug that shortens treatment from 9–12 to 6 months, include the MGIT 960 or BacT/ALERT systems at pH 6.0 (16); both tests are unreliable and subject to frequent false resistance problem and thus not routinely performed (17–20).

To expedite the process of antimicrobial susceptibility testing, we are introducing a novel approach using SYBR Green I/propidium iodide (PI) staining viability assay, which we initially developed for quantifying the viability of and drug evaluation for *Borrelia burgdorferi* (21). The SYBR Green I dye is commonly used in molecular biology to stain nucleic acids and was used for viability assessment of bacteria by flow cytometry (22) but has not been used for AST. SYBR Green I is a permeable dye that stains all live cells green, whereas PI is an impermeant dye that stains only dead or damaged cells red with a compromised cell membrane (21). Hence, the green/red fluorescence ratio as easily measured by fluorescence microscopy or fluorescence microplate readers represents the viability of a bacterial population. As our assay is not growth-dependent, we have eliminated a bottleneck in the current AST.

Here, we demonstrate that the SYBR Green I/PI assay adapted from our previous study (21) can successfully be used for extremely rapid AST for antibiotic-resistant bacteria of different species in 30 min for fast-growing organisms including multidrug-resistant *Staphylococcus aureus, Escherichia coli, Acinetobacter baumannii*, and *Klebsiella pneumoniae*, and in 16 h for slow growing *My. tuberculosis*, producing susceptibility results in concordance with traditional AST methods as provided by the CLSI guidelines. The SYBR Green I/PI assay overcomes not only the inefficiency of conventional culture or growth-based DST but also the limitations and compromised sensitivity of molecular testing methods that rely on detection of mutations that are associated with resistance (23).

## Materials and Methods

### Culture Media, Chemicals, and Antibiotics

*Staphylococcus aureus, E. coli, K. pneumoniae, A. baumannii* cultures were cultivated in tryptic soy broth and tryptic soy agar (Becton Dickinson) and *M. tuberculosis* cultures were cultivated in 7H9 medium at 37°C with the appropriate antibiotics. *S. aureus* strains USA300, CA-409, NY315, CA127, *E. coli* strains W3110, UTI89, CFT073, and KTE181, *K. pneumoniae* (Isolate 7), *A. baumannii* isolate, and *M. tuberculosis* H37Ra, were obtained from ATCC (Manassas, VA, USA). Stock solutions of the antibiotics ampicillin, chloramphenicol, gentamicin, erythromycin, trimethoprim, ciprofloxacin, streptomycin, ceftriaxone, and cefotaxime (Sigma-Aldrich Co.) were prepared and sterilized through filtration and used at indicated concentrations. Antibiotic susceptibility assays for different bacteria were performed using Mueller–Hinton broth and agar per CLSI methods as described below.

### Conventional Antibiotic Susceptibility Tests

The Kirby–Bauer disk diffusion assay was used to determine the susceptibility of the various strains to antibiotics. Instructions and also interpretation of zone diameters were made using the established standards as listed in the Clinical and Laboratory Standards Institute (10). Briefly, bacterial cultures were adjusted to a 0.5 McFarland turbidity standard and spread onto Mueller–Hinton agar. Paper disks were impregnated onto the agar and the antibiotics at concentrations recommended by CLSI were added. The plates were incubated at 37°C overnight before the zone of inhibition was measured. The protocol used to test for antibiotic susceptibility using a broth dilution method was based on the recommendations of CLSI. Briefly, bacterial cultures were adjusted to a 0.5 McFarland turbidity standard in Mueller–Hinton broth and drug concentrations of twofold dilutions.

### SYBR Green I/PI Assay

SYBR Green I (10,000× stock, Invitrogen) was mixed with PI (20 mM, Sigma) in distilled H_2_O. The staining dye for *S. aureus, E. coli, A. baumannii*, and *M. tuberculosis* were made by mixing SYBR Green I to PI (1:3) in 100 µl distilled H_2_O (21). The staining dye for *K. pneumoniae* was made by mixing SYBR Green I to PI (3:1) in 100 µl distilled H_2_O. The SYBR Green I/PI staining mix (10 µl) was added to each 100 µl of each sample. The sample was vortexed and incubated at room temperature in the dark for 20 min. The green and red fluorescence intensity was detected using a Synergy H1 microplate reader by BioTek Instruments (VT, USA) at excitation wavelength of 485 nm and 538 nm and 612 nm for green and red emission, respectively. The percentage of live cells in each sample was determined by a regression equation generated by a standard curve. To generate a standard curve, different proportions of live and isopropyl alcohol killed cells were made. The staining mixture was added to each sample and the green/red fluorescent ratios were measured as described above. The regression equation was generated using the least-square fitting analysis. Specimens of *S. aureus* and *K. pneumoniae* were examined on the Keyence BZ-X710 Fluorescence Microscope and images were recorded and processed using BZ-X Analyzer provided by Keyence (Osaka, Japan). Specimens of *M. tuberculosis* cells were examined on a Zeiss AxioImager M2 microscope equipped with epifluorescence illumination. Pictures were taken using ORCA-R^2^ high resolution digital camera (HAMAMATSU, Japan). Image Pro-Plus software was used to quantitatively determine the fluorescence intensity.

### Isolation of Spontaneous Isoniazid-Resistant and PZA-Resistant Mutants of *M. tuberculosis*

*Mycobacterium tuberculosis* H37Ra was grown in 7H9 liquid medium (Difco) supplemented with 0.05% Tween 80 and 10% bovine serum albumin–dextrose–catalase (ADC) enrichment at 37°C for approximately 10–14 days (mid- to late-log phase). PZA (Sigma-Aldrich Co.) or isoniazid (INH) was dissolved in deionized water at a stock concentration of 10 mg/ml and filter-sterilized and incorporated into 7H11 agar plates containing ADC at concentrations of 100–300 µg/ml, pH 6.0 for PZA, or at 0.2–2 µg/ml for INH. Mutants that grew on the PZA or INH containing plates after 3- to 4-week incubation at 37°C were picked and grown in 7H9 liquid medium to confirm for resistance. The PZA susceptibility testing of the PZA-resistant mutants was performed on 7H11 agar plates containing 100–300 µg/ml PZA (pH 6.0) as described (24).

### SYBR Green I/PI Viability Staining for Rapid Antibiotic Susceptibility Testing

Cultures of *S. aureus, E. coli*, and *K. pneumoniae* were grown to stationary phase and diluted OD600 of 0.1 (approx. 5 × 10^7^) before treatment with antibiotics of indicated concentrations. After 30 min of incubation at 37°C, the cultures were stained with SYBR Green I/PI and the percentage of viable cells was determined as described above. For rapid drug susceptibility testing of *M. tuberculosis*, the culture of parental strain H37Ra and INH-resistant mutant strains were, respectively, added into a 96-well plate, each well containing 80 µl of culture. INH in 20 µl 7H9 medium was added into 80 µl culture as the INH-treated group; meanwhile, the untreated group was the control. The plate was sealed by aluminum seal foil and incubated at 37°C overnight (15 h), followed by the addition of 10 µl SYBR green I/PI staining mixture to the above treated and untreated cultures. The plate was incubated at room temperature in the dark for 45 min followed by fluorescence detection with a microplate reader as described above. Relative to the untreated group, the viability of INH-treated H37Ra and INH-resistant mutants were calculated according to the green and red fluorescence ratio. The difference of residual viability between H37Ra WT and INH-resistant mutants were compared. For PZA susceptibility testing in *M. tuberculosis*, 100 µl of 20-day-old cultures of *M. tuberculosis* H37Ra and PZA-resistant mutants (approx. 5 × 10^6^) was mixed with 4 µl PZA (50 mg/ml) alone or with enhancer [salicylic acid (SA) (40 µg/ml)]. After overnight incubation, SYBR Green I/PI staining dye was added, incubated at room temperature in the dark for 45 min, and the percentage of viable cells was determined as described above. All untreated groups were used as controls.

### Statistical Analyses

Statistical analyses were performed using two-tailed Student’s *t*-test and two-way ANOVA, where appropriate. Mean differences were considered statistically significant if *p* was <0.05.

The proportion of dead cells with their respective 95% confidence intervals was determined using the results from repeated testing from the SYBR Green I/PI assay. All experiments were performed in triplicates. Analyses were performed using GraphPad Prism and Microsoft Office Excel.

## Results

### SYBR Green I/PI Assay Can Assess the Viability of Various Bacterial Pathogens

**Figure 1:**
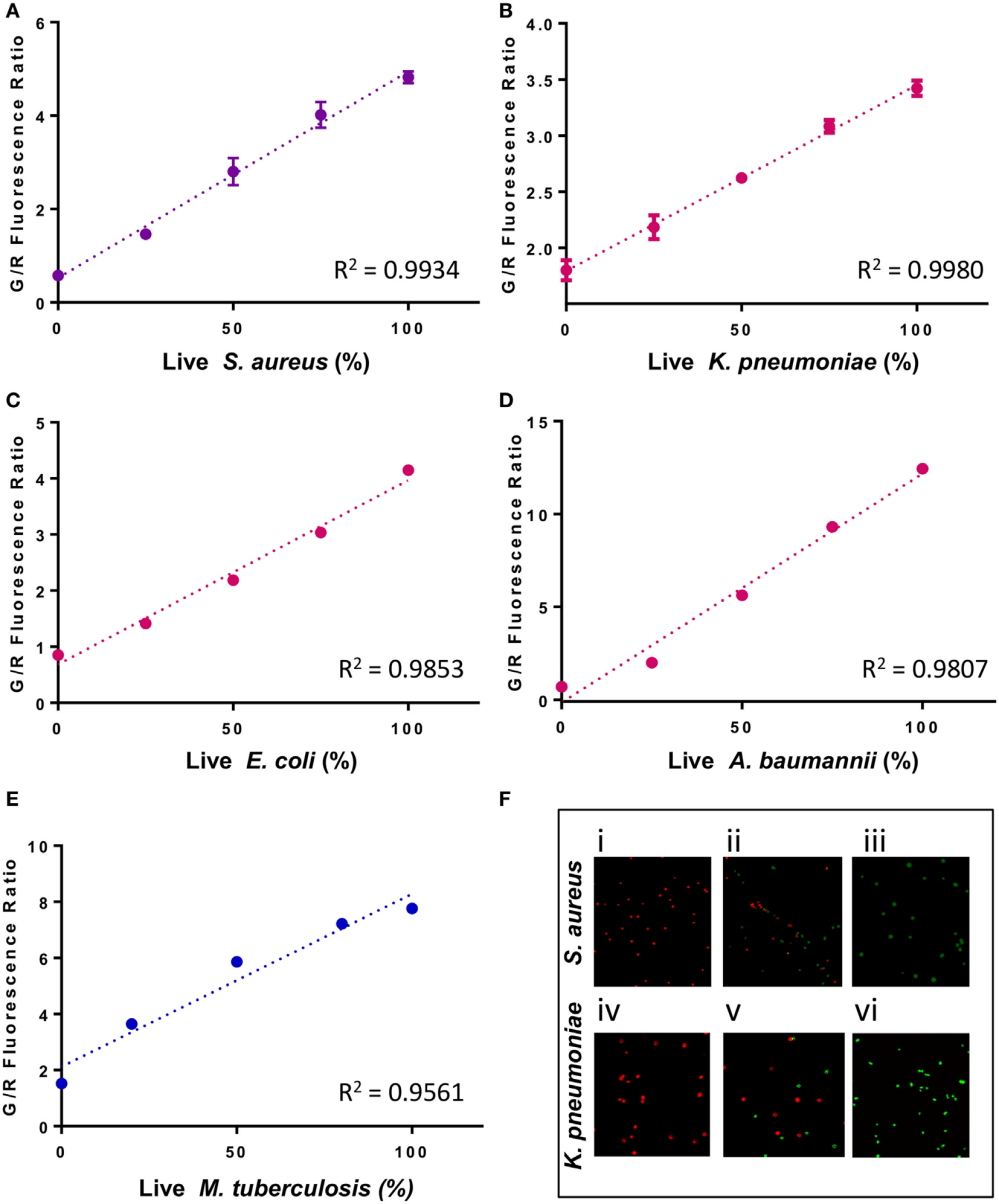
A linear relationship between the percentage of live cells and the green/red fluorescence ratio from the SYBR Green I/propidium iodide (PI) viability assay for different bacterial species. Known proportions of isopropyl killed (30 min) and live **(A)** *Staphylococcus aureus* (USA300), **(B)** *Klebsiella pneumoniae* (Isolate 7), **(C)** *Escherichia coli* (W3110), **(D)** *Acinetobacter baumannii*, and **(E)** *Mycobacterium tuberculosis* (H37Ra) were stained with SYBR Green I/PI and measured using a fluorescence plate reader. A linear regression line was determined. **(F)** Fluorescence microscopy image showing known proportions (0, 50, and 100%) of live (i–iii) *S. aureus* (USA300) and (iv–vi) *K. pneumoniae* (isolate 7) stained with SYBR Green I/PI reveal red (dead) and green (live) ratios in concordance with the known proportions of live and killed organisms from representative Gram-positive (*S. aureus*) and Gram-negative (*K. pneumoniae*) bacteria. Data represent the means ± SEMs.

To develop a rapid test to evaluate antimicrobial susceptibility in the clinic, it is crucial to ensure that SYBR Green I/PI test can assay the viability for a range of test organisms, especially those considered by the CDC as serious or urgent threats. Hence, we applied the SYBR Green I/PI assay to generate a standard curve for representative Gram-positive bacteria *S. aureus*, Gram-negative bacteria *K. pneumoniae, A. baumannii*, and *E. coli*, and acid-fast *M. tuberculosis*. We adapted the SYBR Green I/PI assay which we developed for assessing the viability of *B. burgdorferi* (21) for the above organisms. We grew the bacteria in their normal culture media and to generate dead cells, we killed the bacteria with incubation of 70% isopropyl alcohol for 1 h. After staining the cells with SYBR Green I/PI, we measured the green and red fluorescence intensities using a fluorescence microplate reader (BioTek Synergy HT) and generated a standard curve showing the relationship of bacterial viability and the percentages of green (live) and red (dead) cells. A linear relationship between the green/red fluorescence ratios and the percentages of live *S. aureus, K. pneumoniae, E. coli, A. baumannii*, and *M. tuberculosis* with *R*^2^ values of 0.9934, 0.9980, 0.9853, 0.9807, and 0.9561, respectively, were generated (Figures 1A–E). Additionally, staining of varying viable proportions (0, 50, and 100%) of Gram-positive *S. aureus* (Figure 1F) and Gram-negative *K. pneumoniae* samples (Figure 1F) with SYBR Green I/PI revealed a good correlation between the number of viable cells determined by fluorescence microscopy imaging and the actual known viable percentages determined by the fluorescence microplate reader.

### Development of a Rapid DST Using SYBR Green I/PI Assay for Fast-Growing Organisms

**Figure 2:**
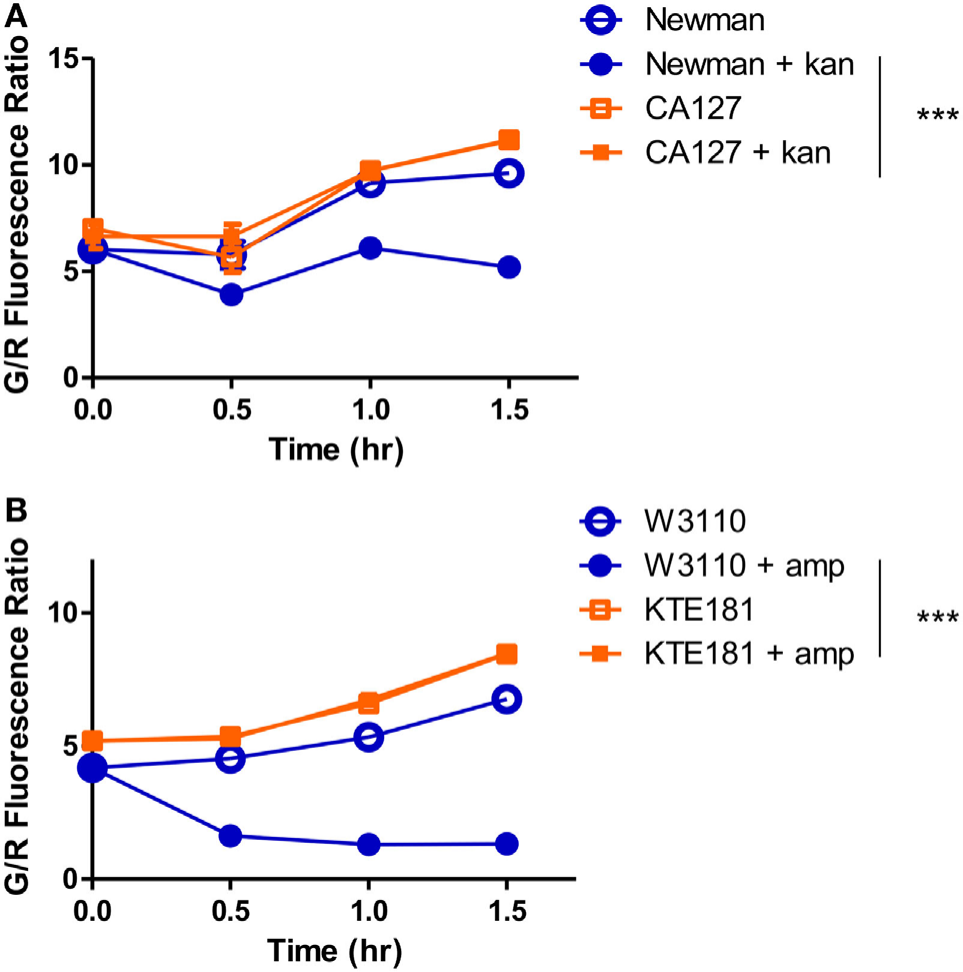
SYBR Green I/propidium iodide monitors the dynamics of resistant and sensitive strains during antibiotic drug exposure. A significant decrease in green/red fluorescence ratio was seen as early as 30 min between **(A)** sensitive *Staphylococcus aureus* strain Newman and resistant strain CA127 during kanamycin exposure and **(B)** sensitive *Escherichia coli* strain W3110 and resistant strain KTE181 during ampicillin exposure (twoway ANOVA, ***p < 0.0005). Data represent the means ± SEMs.

Because resistant organisms are more difficult to kill than susceptible organisms, the SYBR Green I/PI assay can distinguish resistant and susceptible categories based on the amount of residual viable cells after drug treatment (25). Since the SYBR Green I/PI assay detects viability and killing, we hypothesize that this assay will eliminate the time needed for bacterial growth and produce AST results in a shorter time than the current growth-based methods. To test this hypothesis and ensure that our assay produced results that are in concordance with the standards provided by CLSI, we evaluated clinical isolates of different bacterial species for their susceptibility categories against drugs based on the conventional Kirby–Bauer disk diffusion assay (10). Next, to identify an appropriate time where viability differences between resistant and sensitive strains were observed, we measured the green/red fluorescence ratios of both sensitive and resistant strains across 1.5 h of drug exposure time. For *S. aureus*, a significant decrease in viability during kanamycin exposure (100 µg/ml) was observed as early as 30 min between the sensitive Newman strain and the resistant CA127 clinical strain (Figure 2A). For *E. coli*, we also observed a significant decrease in viability in sensitive W3110 strain and resistant KTE181 strain after exposure to ampicillin (100 µg/ml) for 30 min (both, twoway ANOVA, *p* < 0.005) (Figure 2B).

**Figure 3:**
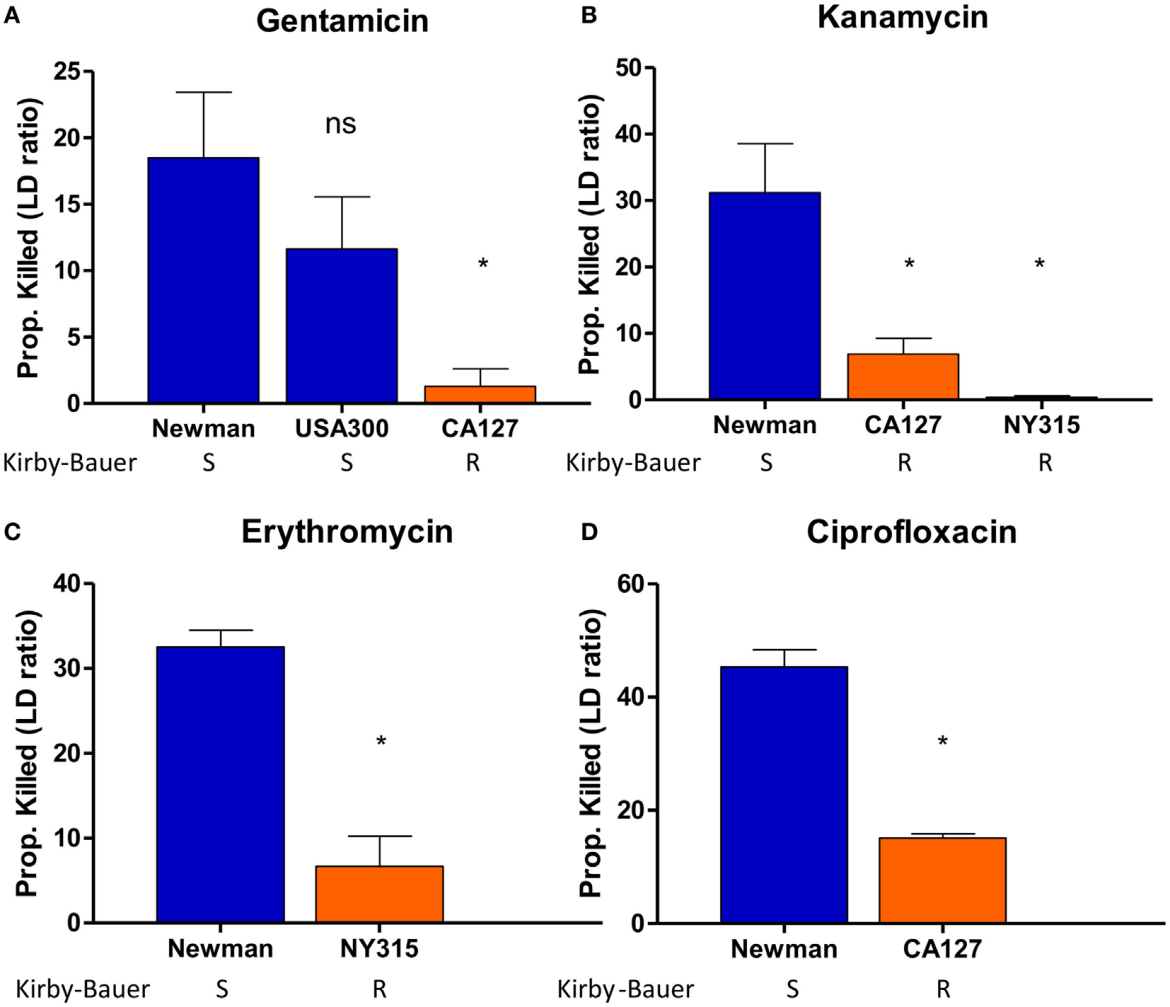
SYBR Green I/propidium iodide (PI) stain can distinguish between R (resistant) and S (sensitive) strains of *Staphylococcus aureus* against various antibiotics in 30 min. Treatment with **(A)** gentamicin (100 µg/ml), **(B)** kanamycin (100 µg/ml), **(C)** erythromycin (400 µg/ml), and **(D)** ciprofloxacin (100 µg/ml) was added to various overnight *S. aureus* strains diluted to 1:25 (OD600 = 0.1). After incubation with antibiotics for 30 min, SYBR Green I/PI staining was performed and distinguished the strains and their respective susceptibility categories. All susceptibility results were in concordance with results from the Kirby–Bauer disk diffusion test (Student’s *t*-test, *p < 0.05). Data represent the means + SEMs.

To optimize the conditions for our SYBR Green/PI assay, we exposed *S. aureus* strains to a panel of bactericidal and bacteriostatic drugs of various concentrations. To establish a quantitative cutoff distinguishing resistant and susceptible strains, we utilized a mathematical formula to determine the proportion of cells killed based on the green and red fluorescence values from microtiter plate readings. The proportion of cells killed was calculated by the formula (LD_treated_ − LD_untreated_)/LD_untreated_, where LD is equal to the ratio of live (green fluorescence) and dead (red fluorescence) cells. We chose a cutoff of approximately 20% of killed cells (between resistant and sensitive strains) which produced statistically significant results (Student’s *t*-test, *p* < 0.05) that were in concordance with susceptibility results based on the interpretive criteria from the conventional Kirby–Bauer method and broth dilution method as published in the CLSI guidelines (Table 1). After 30 min, gentamicin (200 µg/ml) killed 1% of gentamicin-resistant clinical isolate CA127 but 12–26% in sensitive Newman and USA300 strains (Figure 3A). Kanamycin (100 µg/ml) killed 7% of CA127 and 0.5% of NY315 but over 30% of Newman (Figure 3B). Erythromycin (200 µg/ml) killed 2% of NY315 but 26% of sensitive control Newman strain (Figure 3C). Ciprofloxacin (100 µg/ml) killed 15% of CA127 and 45% of Newman strain (Figure 3D). All differences were statistically significant (Student’s *t*-test, *p* < 0.05).

**Table 1:** Summary of broth dilution results and SYBR Green I/propidium iodide (PI) assay.

	Broth dilution CLSI minimum inhibitory concentration (μg/ml)	SYBR Green I/PI Prop.dead avg (95%CI)	CLSI Interpretation	SYBR Green I/Pl Interpretation
***Escherichia coli***				
Ampicillin				
W3110	2	53.9% (40.6–67.1)	S	S
UTI89	2	34.4% (0.08–68.7)	S	S
KTE181	1,024	0% (0–0)	R	R
Trimethoprim				
W3110	1	40.9% (34.8–47.0)	S	S
CFT073	1	27.9% (24.9–31.0)	S	S
KTE181	128	5.9% (2.18–9.76)	R	R

***Staphylococcus aureus***				
Gentamicin				
Newman	4	18.5% (8.81–28.2)	S	S
CA127	16	1.3% (0–3.87)	R	R
Kanamycin				
Newman	8	31.2% (16.7–45.6)	S	S
CA127	16	6.9% (2.32–11.5)	S	S
NV315	128	0.18% (0–0.052)	R	R
Ciprofloxacin				
Newman	0.13	45.3% (39.4–51.3)	S	S
CA127	8	15.1% (13.6–16.6)	R	R

**Figure 4:**
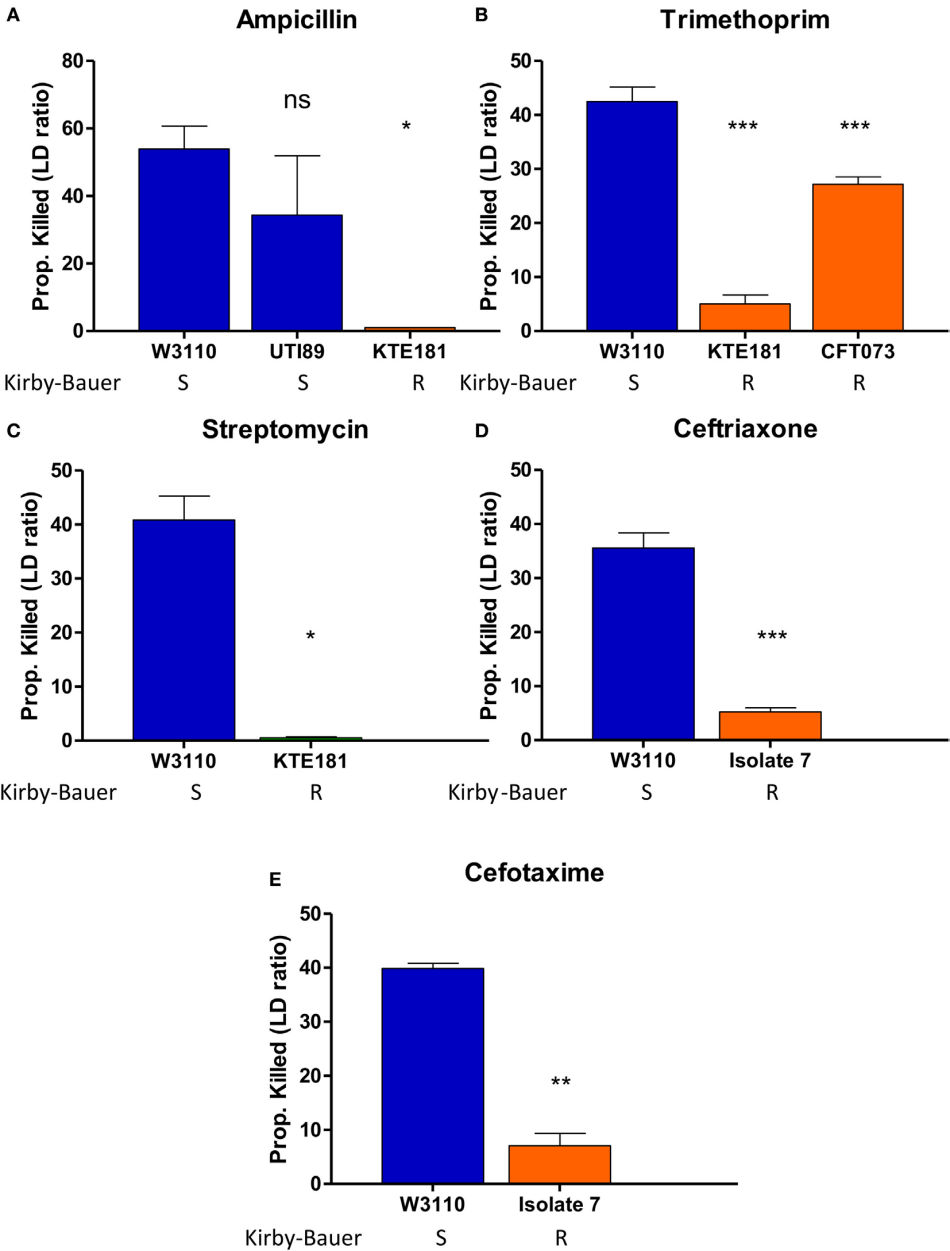
SYBR Green I/propidium iodide (PI) stain can distinguish between R (resistant) and S (sensitive) strains of Gram-negative pathogens against various antibiotics in 30 min. Treatment with **(A)** ampicillin (100 µg/ml), **(B)** trimethoprim (50 µg/ml), and **(C)** streptomycin (50 µg/ml) was added to overnight cultures of *Escherichia coli* strains diluted to 1:25 (OD600 = 0.1). Treatment with **(D)** ceftriaxone (25 µg/ml) and **(E)** cefotaxime (50 µg/ml) was added to overnight cultures of *Klebsiella pneumoniae* strains diluted to 1:25 (OD600 = 0.1). After incubation with antibiotics for 30 min, SYBR Green I/PI staining was performed and distinguished their respective susceptibility categories. All susceptibility results were in concordance with results from the Kirby–Bauer disk diffusion test (Student’s *t*-test, *p < 0.05, **p < 0.05, ***p < 0.0005). Data represent the means + SEMs.

Then, we performed AST for various Gram-negative pathogens including *E. coli* and *K. pneumoniae*. For *E. coli*, upon ampicillin treatment (100 µg/ml), sensitive strains W3110 and UTI89 were killed 53 and 40%, respectively, while resistant strain KTE181 was not killed at all (Figure 4A). Trimethoprim treatment (50 µg/ml) killed 41% of sensitive strain W3110 but 28 and 6% of resistant strains CFT073 and KTE181, respectively (Figure 4B). Streptomycin treatment (50 µg/ml) killed 41% of sensitive strain W3110 but 1% for resistant strain KTE181 (Figure 4C). For *K. pneumoniae* upon ceftriaxone (25 µg/ml) (Figure 4D) and cefotaxime (50 µg/ml) (Figure 4E) treatment, sensitive strain W3110 was killed 38 and 26%, respectively, but the resistant isolate 7 strain was killed 5 and 4%, respectively. All differences were statistically significant (Student’s *t-*test, *p* < 0.05). We did not observe any reaction between antibiotics tested and the SYBR Green I and PI dyes used, and dyes used at the testing concentrations did not significantly affect the viability of the bacteria in control experiments.

### Demonstration that Bacteriostatic Drugs Work as Well as Bactericidal Drugs With SYBR Green I/PI Assay in Distinguishing Sensitive and Resistant Strains

**Figure 5:**
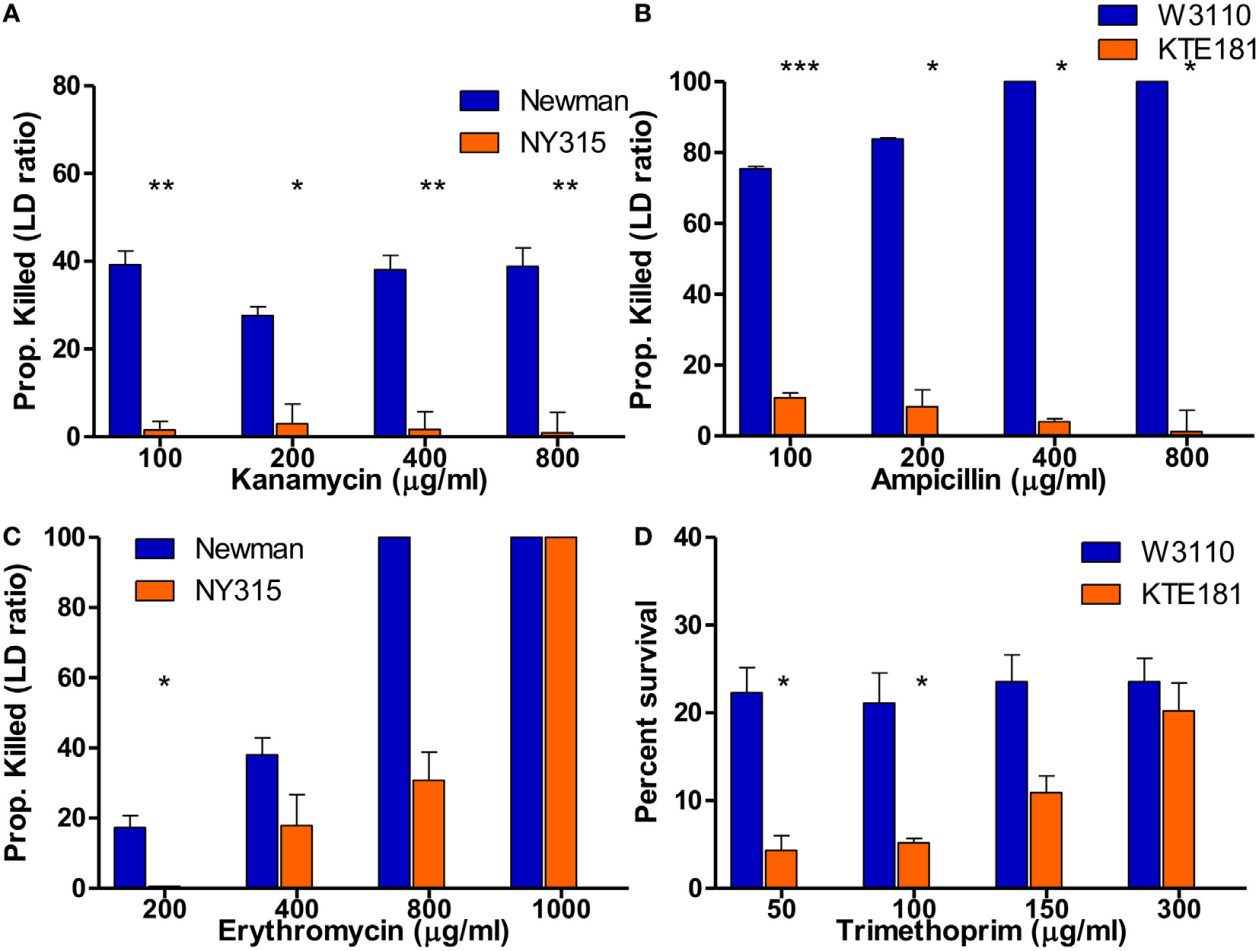
SYBR Green I/propidium iodide (PI) assay reveals killing of bactericidal and bacteriostatic antibiotics in 30 min. SYBR Green I/PI assay reveals killing between sensitive and resistant strains after treatment with increasing concentrations of bactericidal drugs **(A)** kanamycin for *Staphylococcus aureus* and **(B)** ampicillin for *Escherichia coli*. Upon treatment with increasing concentrations of bacteriostatic drugs **(C)** erythromycin for *S. aureus* and **(D)** trimethoprim for *E. coli*, SYBR Green I/PI assay was consistently able to distinguish sensitive and resistant strains when administered at different concentrations for both sensitive and resistant strains (Student’s *t*-test, *p < 0.05, **p < 0.005, ***p < 0.0005). Data represent the means + SEMs.

By definition, bactericidal drugs kill the bacteria whereas bacteriostatic drugs inhibit the growth of the bacteria. Here, we wanted to address if the SYBR Green I/PI assay can detect sensitive and resistant strains of not only bactericidal drugs but bacteriostatic drugs as well. Our SYBR Green I/PI assay revealed that treatment with increasing concentrations of bactericidal drug kanamycin for 30 min consistently killed 40% of the cells in the sensitive *S. aureus* Newman strain but an average of 2% in the resistant NY315 strain, as expected (Figure 5A). For *E. coli*, the percentage of killed cells after treatment with bactericidal drug ampicillin increased in a dose-dependent manner. A statistical significant difference between the sensitive strain W3110 and resistant strain KTE181 was evident using 100 µg/ml of ampicillin (Figure 5B).

To test if the SYBR Green I/PI assay can detect sensitive and resistant strains by bacteriostatic drugs, we added increasing concentrations of bacteriostatic agent erythromycin (200–1,000 µg/ml) for *S. aureus* strains (Figure 5C) and bacteriostatic drug trimethoprim (50–300 µg/ml) for *E. coli* strains (Figure 5D). In both cases, the proportion of dead cells (measured by green/red fluorescence ratio) increased in a dose-dependent manner as determined by the SYBR Green I/PI staining. For both sensitive and resistant strains, all cells were killed as the concentrations of drugs increased. Significant difference between the amount of killed cells in sensitive *S. aureus* Newman and resistant NY315 strain (using 200 µg/ml erythromycin) and sensitive *E. coli* W3110 and resistant KTE181 strain (using 50 µg/ml trimethoprim) were observed at appropriate drug concentrations, though killing activity between the strains became insignificant when the highest concentration of drugs [erythromycin (>400 µg/ml) and trimethoprim (>150 µg/ml)] was administered.

### Implementation of SYBR Green I/PI Assay for Rapid Drug Susceptibility Testing for *M. tuberculosis*

**Figure 6:**
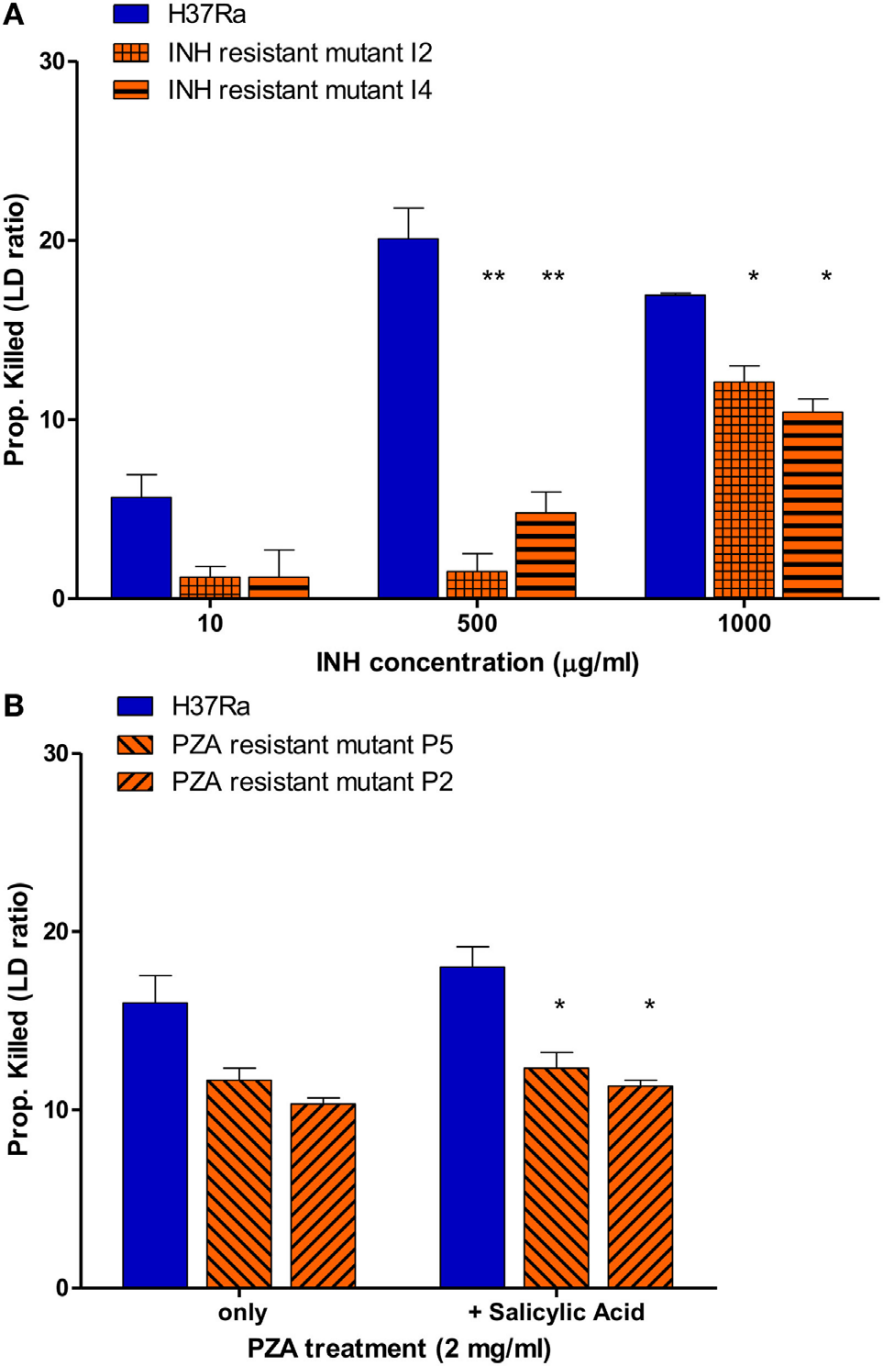
Drug susceptibility testing of *Mycobacterium tuberculosis* against first-line tuberculosis drugs using the SYBR Green I/propidium iodide (PI) assay. **(A)** *M. tuberculosis* H37Ra and INH-resistant mutants I2, I4 (10-day old) were treated with 10, 500, and 1,000 µg/ml INH overnight (16 h). INH-resistant mutants I2 and I4 were determined to be resistant using 500 and 1,000 µg/ml INH. **(B)** *M. tuberculosis* parental strain H37Ra and pyrazinamide (PZA)-resistant mutants (P5 and P2) (20-day old) were treated with PZA (2 mg/ml) overnight (16 h). Treatment with salicylic acid (40 µg/ml) strongly increased the efficacy SYBR Green I/PI assay in detecting PZA resistance in mutants P5 and P2 compared to parental strain H37Ra (Student’s *t*-test, *p < 0.05, **p < 0.005). Data represent the means + SEMs.

To determine if this assay also works for slow growing *M. tuberculosis*, we tested the utility of the SYBR Green/PI assay for detecting isoniazid (INH) resistance. Because INH mainly acts on growing cells of *M. tuberculosis* (23) we used a 7- to 10-day log phase culture of *M. tuberculosis* H37Ra and INH-resistant mutants (I2 and I4). We treated parental strain H37Ra and resistant mutants with different INH concentrations for 16 h, followed by the SYBR Green I/PI assay to determine the percentage of cells killed. No significant differences between the proportion of killed H37Ra and INH-resistant strains at INH concentration of 10 µg/ml were observed. However, at higher concentrations of INH (500 and 1,000 µg/ml), parental strain H37Ra was killed more effectively compared to its resistant mutants I2 and I4 (Figure 6A) (*p* < 0.005, *p* < 0.05, respectively). A high concentration of INH (1,000 µg/ml), also caused death of the INH-resistant strains reducing the difference between the sensitive parent strain and the resistant mutants (Figure 6A). These results indicate that the SYBR Green I/PI assay at the appropriate drug concentration (500 µg/ml) can rapidly detect INH resistance in *M. tuberculosis* in 16 h.

We then went on to assess if the SYBR Green/PI assay could be used for detecting PZA resistance, the most challenging DST of all TB drugs (17–19). Because PZA plays a critical role in shortening TB treatment from 9–12 to 6 months and is used for treating both drug susceptible and drug-resistant TB, accurate testing of PZA susceptibility is crucial for treatment outcome (15, 26). Unfortunately, the current PZA susceptibility testing methods often produce unreliable false positive results and can take weeks due to reliance on growth of *M. tuberculosis* (17, 19, 20). Also, a small percentage of PZA-resistant strains do not have mutations in *pncA* or *rpsA* involved in PZA’s mechanisms of resistance, which limits the capacity of molecular methods for detecting PZA resistance (27). To evaluate if our rapid AST can detect PZA resistance, we treated parental strain *M. tuberculosis* H37Ra (MIC < 100 μg/ml PZA, pH 5.9) and PZA-resistant mutants (>200 μg/ml PZA, pH 5.9) with different PZA concentrations followed by SYBR Green I/PI staining. We found that the difference in proportion of dead cells between the susceptible H37Ra and PZA-resistant strains increased with higher PZA concentration after overnight (16 h) treatment. After optimization, we found that susceptible H37Ra strain was killed more effectively compared to PZA-resistant mutants with 2 mg/ml PZA treatment overnight (Figure 6B), suggesting that our assay could identify PZA resistance in just 16 h.

Since PZA acts differently from common antibiotics by killing non-replicating *M. tuberculosis* persisters (27), certain weak acids such as SA and acid pH are known to enhance the activity of PZA against *M. tuberculosis* (28, 29). Therefore, we tested if weak acid SA added to the PZA treatment could show more significant difference in residual viability between the parental susceptible strain H37Ra and PZA-resistant mutants. Indeed, we found that SA (40 µg/ml) significantly enhanced the activity of PZA against parental strain H37Ra but not PZA-resistant mutants at 2 mg/ml PZA in overnight (16 h) treatment (Figure 6B). The proportion of dead H37Ra was significantly higher than the two PZA-resistant mutants P2 and P5 (Figure 6B) (*p* < 0.05). These results showed that the SYBR Green I/PI assay with weak acids could differentiate PZA susceptible and PZA-resistant strains more clearly than in their absence, allowing for more quantitative measurements of PZA resistance in *M. tuberculosis*.

## Discussion

The current conventional AST relies on the growth of the test organism in the presence of the antibiotic, which can be time-consuming depending on the growth speed of the organism, ranging from days for fast-growing bacteria to weeks for slow growing bacteria such as *M. tuberculosis*. Here, we developed a rapid and novel antibiotic susceptibility testing methodology using SYBR Green I/PI assay which converts a conventional growth/culture-dependent AST to a viability-based growth-independent AST. This study is the first to demonstrate the feasibility of the adapted SYBR Green I/PI viability assay to rapidly and reliably detect antibiotic resistance for representative Gram-positive *S. aureus*, Gram-negative bacteria *E. coli, K. pneumoniae* in record time of just 30 min, and identify INH and PZA resistance for slow-growing *M. tuberculosis* in just 16 h, which currently takes several weeks. Thus, our newly developed methodology changes the concept of the current antibiotic susceptibility testing and significantly reduces the time needed for the current growth-dependent AST.

The automated BACTEC™ MGIT™ 960 (Becton Dickinson, Sparks, MD, USA) is the most commonly used system for *M. tuberuclosis* DST and takes 1–3 weeks, while the Lowenstein–Jensen and 7H10/11 solid media-based DST can take 3–6 weeks, due to the slow growth of the organism. While previous studies have shown that flow cytometry can be used for AST testing for *M. tuberculosis* in 24 h (30), the test still relies on growth detection by fluorescence diacetate and has significant drawbacks such as the requirement for expensive instrumentation and lack of high-throughput, and as a result, such method has not been adopted for DST for *M. tuberculosis* in clinical settings. Despite the high sensitivity achieved by the MBT-ASTRA, as the authors noted, it seems unlikely that MBT-ASTRA will be used in clinical laboratories due to its lack of automated readout (12). Additionally, a luciferase phage system which was developed to detect rifampicin resistance in mycobacteria in 2 days has contamination issues (31). In contrast, the SYBR Green I/PI assay we developed can be used for ultra-rapid DST for not only fast-growing organisms but also for slow-growing organisms like *M. tuberculosis* in a high-throughput manner such as in 96-well or 384-well plate format, has low detection limits, background noise and more quantitative, and is extremely cost-effective.

It is worth noting that our SYBR Green/PI methodology is different from the current growth-dependent MIC-based AST in that our test used concentrations of antibiotics that are much (10- to 10,000-fold) higher than the MIC values and are chosen based on their ability to distinguish susceptible and resistant strains of the bacterial species tested. We acknowledge that such high concentrations of drugs do not correlate with Cmax values, but such high drug concentrations allow for extremely rapid determination of susceptible versus resistant strains in a record short time. While resistant strains can also be killed in high concentrations of drug, strains KTE181 (*E. coli*) and NY315 (*S. aureus)* were characterized as resistant to ampicillin and kanamycin, respectively, by CLSI guidelines showed an average of 0% of death in our SYBR Green I/PI assay suggesting that higher concentrations of drug used may not always result in killing (Table 1).

The start of an *in vitro*-cultivation is often associated with the lag phase as adaptation phase of the bacteria to changed growth conditions. This phenomenon could influence the killing of the bacteria by antibiotics and, therefore, the green/red fluorescence ratio. Nevertheless, we were still able to detect a greater proportion of dead cells in our susceptible strains for all the organisms tested, presumably due to the high concentrations of antibiotics used. If certain antibiotic does not show good killing on the diluted culture directly, we could potentially enhance the metabolic status of the bacteria by allowing the bacteria to grow for a short time or by adding factors that increase metabolic activity of the bacteria so they respond or become more susceptible to killing by the antibiotic.

It is important to note that our SYBR Green I/PI assay works with several major classes of antibiotics that have different modes of action including inhibitors of cell wall, protein synthesis, DNA gyrase and anti-metabolite agents, and even drugs with unusual mechanisms of action such as PZA. Moreover, our SYBR Green I/PI assay not only works for bactericidal antibiotics (e.g., ampicillin, streptomycin, ciprofloxacin, gentamicin, and kanamycin) but also for bacteriostatic drugs (e.g., trimethoprim, erythromycin, and chloramphenicol) (Figures 2–4). Most importantly for *M. tuberculosis*, resistance to the critical persister drug PZA, which has been very difficult to determine with current time-consuming growth-based methods, such as MGIT960 (32), can be easily and accurately detected by our SYBR Green/PI viability assay in just 16 h. Resistance to drugs, such as rifampin, isoniazid, linezolid, and ethambutol, was evaluated in newer MALDI-TOF DST methods but PZA, a first-line drug for treating tuberculosis, has been ignored (12). In fact, the SYBR Green/PI based assay is more appropriate and advantageous for determining the susceptibility of slow growing *M. tuberculosis* to persister drugs like PZA than the conventional MIC-based DST as PZA is mainly active against non-growing persisters with little activity against growing bacilli (27).

There is an increasing antibiotic resistance crisis ongoing and new drugs and antibiotics are urgently needed to combat the life threatening antibiotic-resistant infections. Our newly developed SYBR Green I/PI AST testing methodology, which we developed initially for rapid assessment of viability of *B. burgdorferi* (21) and subsequently used for high-throughput drug screens against fastidious organism *B. burgdorferi* persisters (25), can also be adapted for efficient and versatile real-time continuous drug screens to identify new drug candidates targeting both growing and non-growing antibiotic-resistant bacteria, eukaryotic pathogens, and cancer in a high-throughput format.

Despite the potential for the very rapid detection of antibiotic resistance, we are fully aware that future studies to test more clinical strains with different drugs are needed to validate the new SYBR Green I/PI method before implementation in the clinic. Future studies include conducting retrospective and prospective studies using a larger collection of strains to validate our breakpoints in determining the different susceptibility categories as characterized in the CLSI guidelines (e.g., resistant, intermediate resistant, and susceptible). Thus, stronger associations between the proportion of dead cells (given by our SYBR Green I/PI assay) and the MIC of a particular drug (from broth dilution methods) can be made so that clinicians can extract more useful clinical information from our assay. However, it is important to note that based on MIC breakpoints from CLSI, *S. aureus* strains Newman and CA127 are both classified as susceptible to kanamycin despite CA127 having a higher MIC than Newman, 16 and 8 µg/ml, respectively. Our SYBR Green I/PI assay was sensitive enough to detect such difference given that the proportion of dead cells in CA127 was lower than Newman, 6.9 and 31.2%, respectively. Additionally, the 95% confidence interval of resistant and susceptible strains of each particular species do not overlap suggesting that our assay conditions are robust enough to potentially detect differences in MIC.

The goal of our study is to provide a proof of concept of using SYBR Green/PI as a novel and rapid method for detection of antibiotic resistance. In this study, we showed that the SYBR Green/PI assay can be used for this purpose. Although our studies are on select representative Gram-positive, Gram-negative bacteria, and mycobacteria, this methodology can be adapted to detect resistance in all culturable organisms of prokaryotic and eukaryotic origin for both growing and non-growing cells for both drug susceptibility testing and drug screens in a real-time manner. We anticipate that this new test, subject to further clinical validation, may play important roles in saving lives and improving patient outcomes in a timely manner, as well as identify new drugs for improved treatment of antibiotic-resistant organisms.

## Author Contributions

JF and RY contributed equally to this work. YZ, JF, RY, and W-HZ designed the study. JF, RY, SZ, LT, and WS performed the study. JF, RY, W-HZ, and YZ analyzed the data. RY, JF, and YZ wrote the manuscript.

## Conflict of Interest Statement

A patent application (# US 15/347,285, EP17159022.7-1405, CN201580037580.4) pertaining to the results presented in the paper was filed.
